# Supplementary material for: Temperature-dependent modulation of odor-dependent behavior in three drosophilid fly species of differing thermal preference
Source: Commun Biol. 2023 Sep 4;6:905. doi: 10.1038/s42003-023-05280-5 (PMC10477191; doi:10.1038/s42003-023-05280-5)
Supplement: Supplementary file 1 — Supplementary Information [file 42003_2023_5280_MOESM1_ESM.pdf]

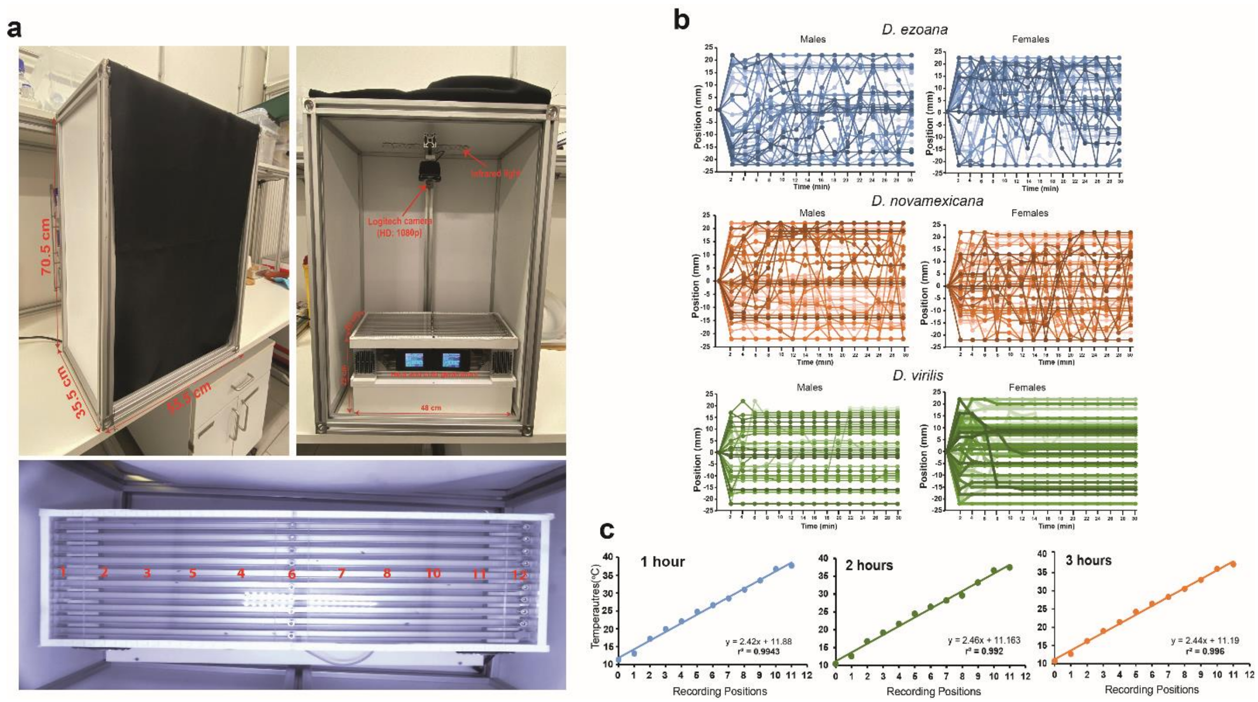

Supplementary figure 1. **Experimental setup for testing temperature preference in the three drosophilid flies.** **(a)** Thermal gradient apparatus, **(b)** Tracking lines showing the longitudinal position (recorded every 2 min) of *D. ezoana*, *D. novamexicana*, and *D. virilis* within the aluminium channels in absence of a thermal gradient. **(c)** Line graphs depicting the increase of temperature in the aluminium channels (from the cold region to the hot region) 1, 2 and 3 hours after turning on the heat and cold generators. Each dot on the graphs represents the temperature of a specific region of the aluminium channels.

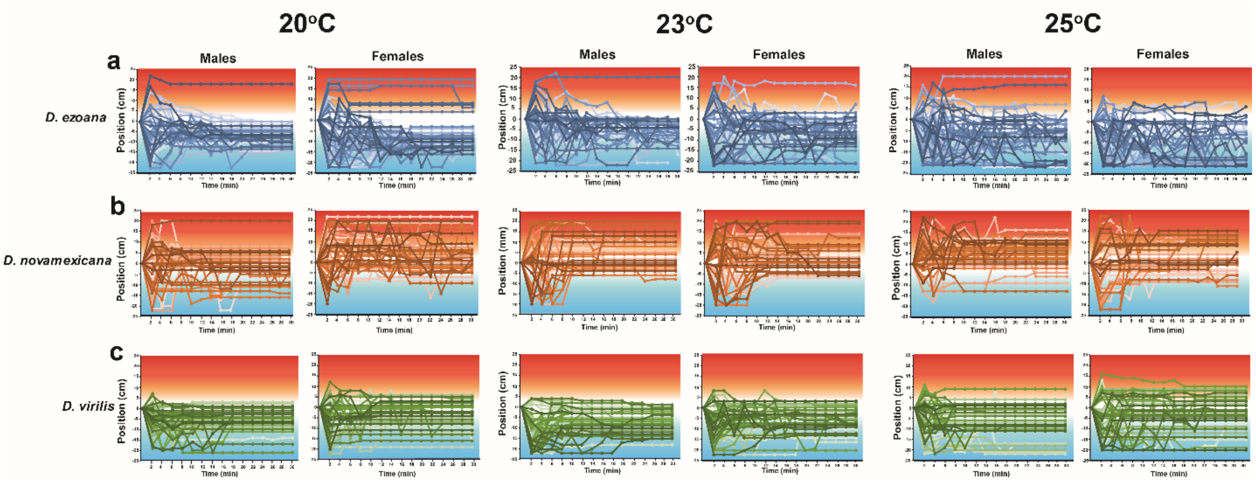

Supplementary figure 2. Locomotion patterns in the three drosophilid species after the establishment of the thermal gradient. Tracking lines showing the longitudinal position (recorded every 2 min) of **(a)** *D. ezoana*, **(b)** *D. novamexicana*, and **(c)** *D. virilis* individuals reared at 20ºC (left), 23ºC (middle) and 25 ºC right) and placed within the aluminium channels in presence of the thermal gradient.

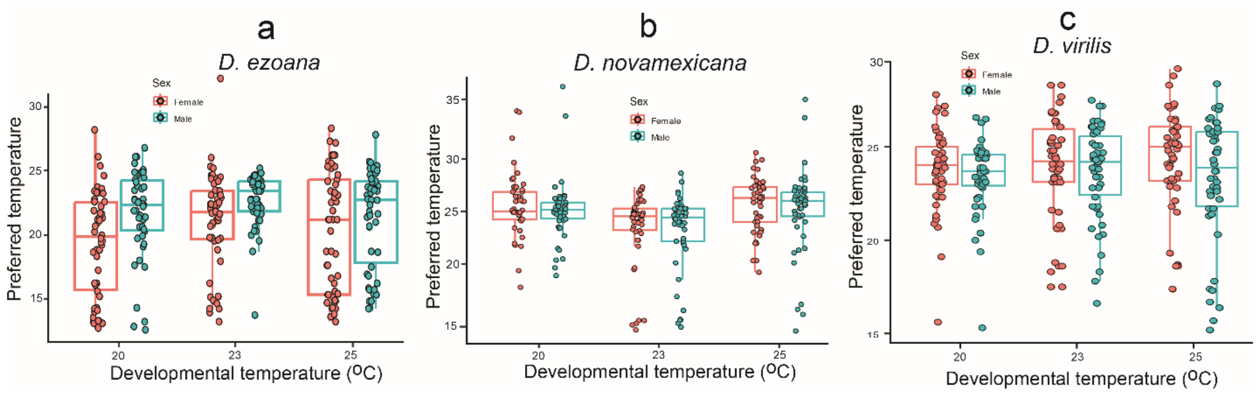

Supplementary figure 3. Temperature preference of the three *Drosophila* species. Boxplot illustrating the preferred temperatures of males and females of **(a)** *D. ezoana*, **(b)** *D. novamexicana* and **(c)** *D. virilis* when reared at 20 ºC , 23ºC and 25 ºC. Boxplot whiskers indicate ± 1.5 interquartile range limits. Dots in each boxplot indicate individual temperature data points (n=50 biologically independent samples).

Supplementary table 1: Three-way analysis of variance table for the effect of developmental temperature, sex and species on temperature preference in the three Drosophilid species

Factors	DF	Sum Sq	Mean Sq	F value	Pr(>F)
Developmental Temperature	2	29	14.3	1.282	0.278
Species	2	2469	1234.6	110.818	***P<0.0001***
Sex	1	10	10	0.901	0.343
Developmental Temperature × Species	4	431	107.9	9.682	***P<0.0001***
Developmental Temperature ×Sex	2	21	10.4	0.937	0.392
Species × Sex	2	259	129.6	11.631	***P<0.0001***
Developmental Temperature × Species × Sex	4	13	3.3	0.298	0.88

Number in bold/italic depict significant differences

Supplementary table 2. List of odorants used in the electrophysiology experiment

	Compound names	CAS number		Compound names	CAS number
1	Ethyl acetate	141-78-6	30	Isopentyl alcohol	123-51-3
2	Ethyl lactate	97-64-3	31	1-hexanol	111-27-3
3	Methyl salicylate	119-36-8	32	2-methyl phenol	95-48-7
4	Methyl acetate	79-20-9	33	2-nonanone	821-55-6
5	Ethyl-3-hydroxybutyrate	5405-41-4	34	Isopentyl acetate	123-92-2
6	ethyl hexanoate	123-66-0	35	4-methylphenol	106-44-5
7	2-heptanone	110-43-0	36	Acetophenone	98-86-2
8	E2-hexanal	6728-26-3	37	methyl hexanoate	106-70-7
9	geosmin	16423-19-1	38	propyl acetate	109-60-4
10	geranyl acetate	105-87-3	39	citral	5392-40-5
11	pentyl acetate	628-63-7	40	2,3-Butanediol	513-85-9
12	1-octen-3-ol	3391-86-4	41	3-hexanone	589-38-8
13	guiacol	90-05-1	42	2-hexanone	591-78-6
14	ethyl benzoate	93-89-0	43	3-hexanol	623-37-0
15	Ethyl crotonate	623-70-1	44	2-hexanol	626-93-7
16	acetoin	513-86-0	45	nonanal	124-19-6
17	linalool	5392-40-5	46	2,6-dimethoxy-phenol	91-10-1
18	2 phenyalcohol	60-12-8	47	3,4,5-Trimethoxyphenol	642-71-7
19	benzyl butyrate	103-37-7	48	Tetradecane	629-59-4
20	2-butanone	78-93-3	49	9-Octadecen-1-ol, (Z)-	143-28-2
21	ethyl butanoate	105-54-4	50	Oleic acid	112-80-1
22	isopropyl benzoate	939-48-0	51	(Z)-methyl ester 9-Octadecenoic acid	112-62-9
23	Dimethyl disulphide	624-92-0	52	oleamide	301-02-0
24	acetone	67-64-1	53	4-Ethylguaiacol	2785-89-9
25	methyl benzoate	93-58-3	54	phenol	108-95-2
26	6-methyl-5-helpten-2-one	110-93-0	55	Acetovanillone	498-02-2
27	Hexyl acetate	142-92-7	56	Salicylaldehyde	90-02-8
28	Isopentyl propionate	105-68-0	57	4 -ethylphenol	123-07-9
29	2-nonanol	628-99-9	58	hexadecanamide	629-54-9

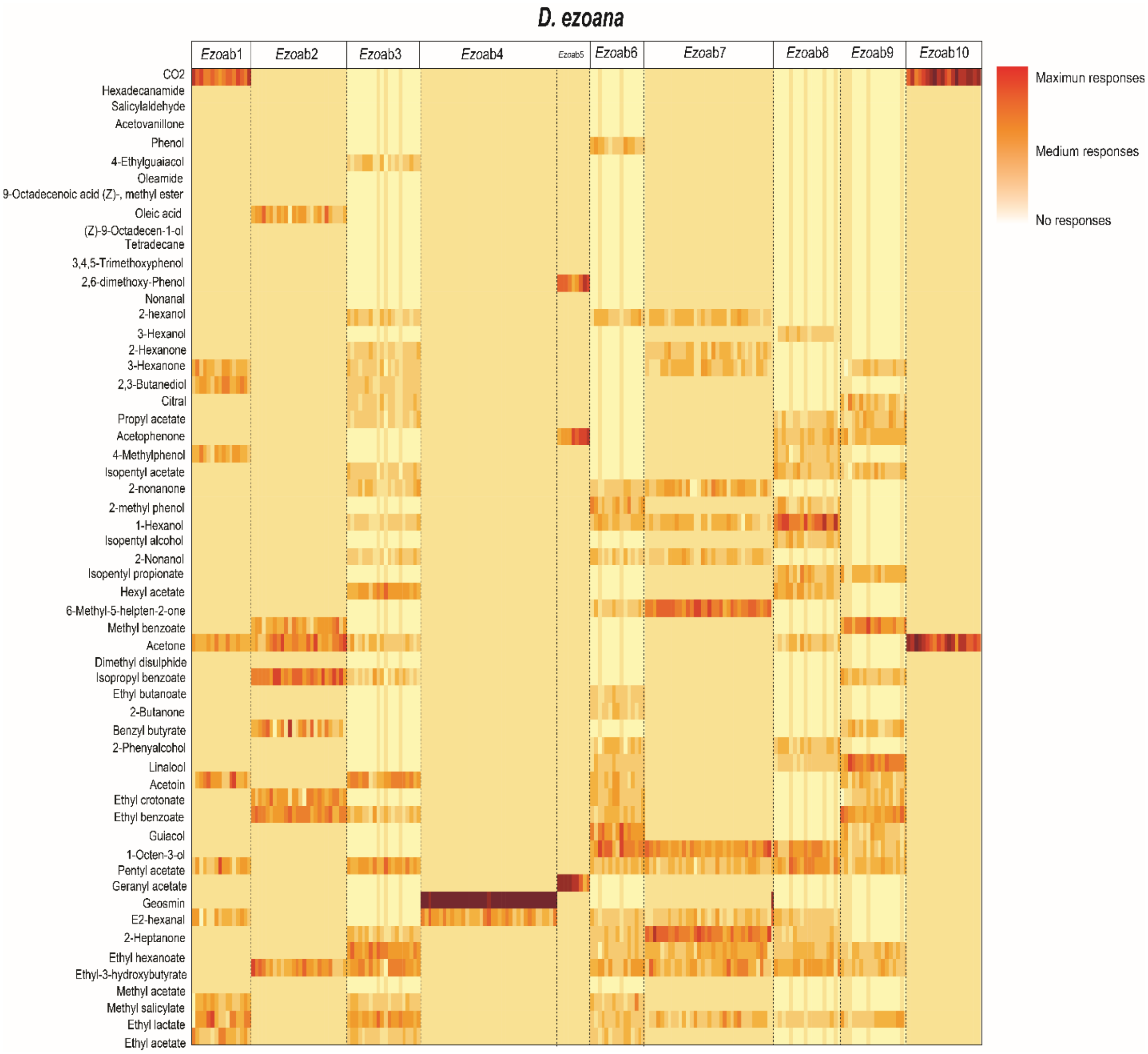

Supplementary figure 4. **Functional classification of the antennal basiconic sensilla of *Drosophila ezoana*.** Heat map shows the response profiles of the 200 individual basiconics stimulated with the 57-odorant panels. Red indicates a maximum response; dark-orange a medium response; and yellow no response.

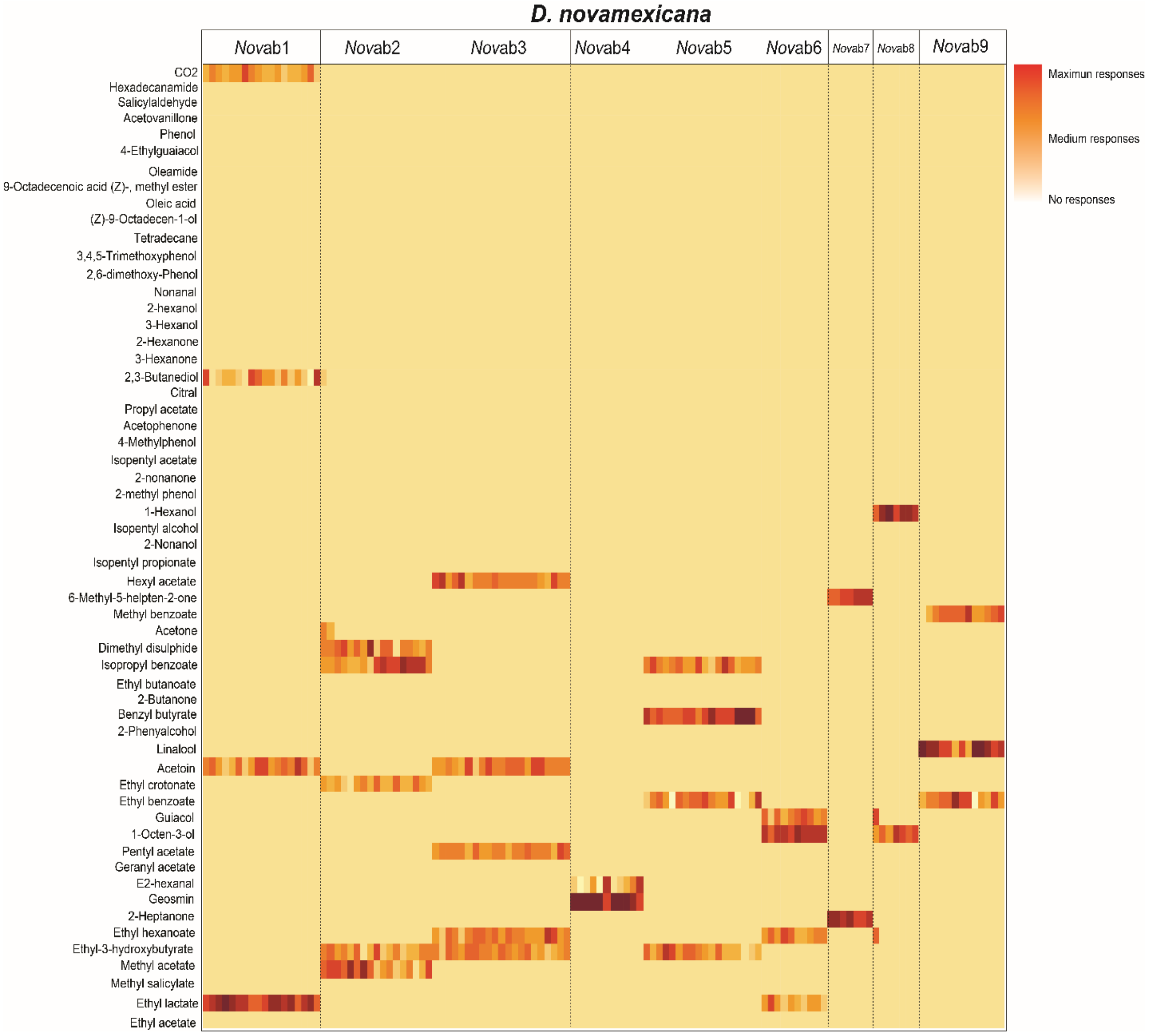

Supplementary figure 5. Functional classification of the antennal basiconic sensilla of *Drosophila novamexicana*. Heat map shows the response profiles of the 200 individual basiconics stimulated with the 57-odorant panels. Red indicates a maximum response; dark-orange a medium response; and yellow no response.

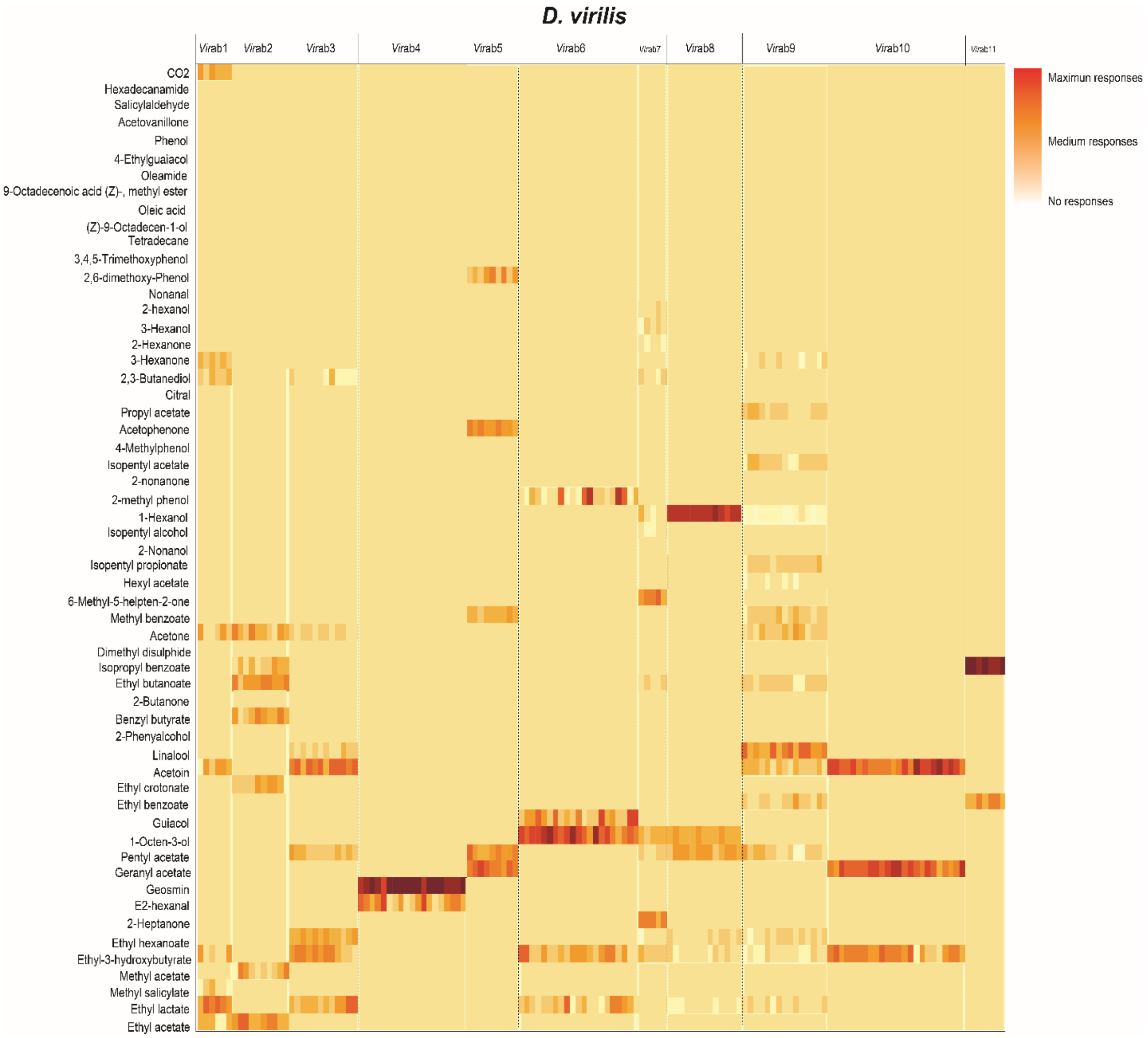

Supplementary figure 6. Functional classification of the antennal basiconic sensilla of *Drosophila virilis*. Heat map shows the response profiles of the 200 individual basiconics stimulated with the 57-odorant panels. Red indicates a maximum response; dark-orange a medium response; and yellow no response.

Supplementary table 3. List of basiconic olfactory sensory neurons found in *Drosophila melanogaster* and their key ligands

	D. melanogaster	
Sensillum types	Neurons	Best ligands
*Mel*ab1	A	Ethyl acetate
	B	Ethyl lactate
	C	CO2
	D	Methyl salicylate
*Mel*ab2	A	Methyl acetate
	B	Ethyl-3-hydroxybutyrate
*Mel*ab3	A	Ethyl hexanoate
	B	2-heptanone
*Mel*ab4	A	E-2-hexanal
	B	Geosmin
*Mel*ab5	A	Geranyl acetate
	B	Pentyl acetate
*Mel*ab6	A	1-octen-3-ol
	B	Guaiacol
*Mel*ab7	A	Ethyl benzoate
	B	Ethyl lactate
*Mel*ab8	A	Ethyl-E-2-butanoate
	B	3-Hydroxy-2-butanone
*Mel*ab9	A	Linalool
	B	2-Phenylethanol
*Mel*ab10	A	Benzyl butyrate
	B	Unknown

Supplementary table 4. List of basiconic olfactory sensory neurons found in *Drosophila ezoana* and their key ligands

			D. ezoana
Sensillum types	Number of sensilla recorded	Neurons	Best ligands
*Ezo*ab1 (like Melab1)	16	A	Acetoin/Aceton
		B	Ethyl lactate/2,3-Butanediol/Pentyl acetate
		C	CO2
		D	Methyl salicylate
*Ezo*ab2	26	A	Acetone/Methyl benzoate/Methyl acetate
		B	Isopropyl benzoate/Ethyl benzoate/Ethyl-3-hydroxybutyrate
*Ezo*ab3	20	A	Acetoin/Ethyl hexanoate/Pentyl acetate/Hexyl acetate
		B	Ethyl lactate/Ethyl-3-hydroxybutyrate
*Ezo*ab4 (like *Mel*ab4)	31	A	E-2-hexanal
		B	Geosmin
*Ezo*ab5	9	A	Geranayl acetate/ Acetophenone
		B	2,6-Dimethoxy-phenol
*Ezo*ab6 (like *Mel*ab6)	15	A	1-octen-3-ol
		B	Guaiacol
*Ezo*ab7	27	A	6-Methyl-5-hepten-2-one/1-octen-3-ol
		B	2-Heptanone
*Ezo*ab8	18	A	1-Hexanol
		B	Isopropyl propionate
*Ezo*ab9	18	A	Linalool
		B	Methyl benzoate
*Ezo*ab10	20	A	Acetone
		B	CO2

Supplementary table 5. List of basiconic olfactory sensory neurons found in *Drosophila novamexicana* and their key ligands

			D. novamexicana
Sensillum types	Number of sensilla recorded	Neurons	Best ligands
*Nov*ab1 (like Melab1)	17	A	Acetoin/Ethyl-3-hydroxybutyrate
		B	Ethyl lactate
		C	CO2
		D	Methyl salicylate
*Nov*ab2	19	A	Methyl acetate/Dimethyl dissulfide
		B	Isopropyl benzoate
*Nov*ab3	28	A	Acetoin/Hexyl acetate/Ethyl hexanoate/Pentyl acetate
		B	Ethyl lactate/Ethyl-3-hydroxybutyrate
*Nov*ab4 (like *Mel*ab4)	21	A	E-2-hexanal
		B	Geosmin
*Nov*ab5	18	A	Benzyl butyrate
		B	Isopropyl benzoate
*Nov*ab6 (like *Mel*ab6)	16	A	1-octen-3-ol
		B	Guaiacol/Ethyl-3-hydroxybutyrate
*Nov*ab7	27	A	6-Methyl-5-hepten-2-one/1-octen-3-ol
		B	2-Heptanone
*Nov*ab8	31	A	1-Hexanol/Pentyl acetate/1-Octen-3-ol
		B	Ethyl lactate
*Nov*ab9	23	A	Linalool
		B	Methyl benzoate

Supplementary table 6. List of basiconic olfactory sensory neurons found in *Drosophila virilis* and their key ligands

			D. virilis
Sensillum types	Number of sensilla recorded	Neurons	Best ligands
*Vir*ab1 (like *Mel*ab1)	6	A	Acetoin/Aceton/3-Hexanone
		B	Ethyl lactate/isopentyl alcohol
		C	CO2
		D	Methyl salicylate
*Vir*ab2	21	A	Ethyl acetate/Acetone/Benzyl butyrate/Methyl acetate
		B	Ethyl butanoate/Ethyl crotonate/Isopropyl benzoate
*Vir*ab3	24	A	Acetoin/Ethyl hexanoate/Pentyl acetate
		B	Ethyl lactate/Ethyl-3-hydroxybutyrate
*Vir*ab4 (like *Mel*ab4)	19	A	E-2-hexanal
		B	Geosmin
*Vir*ab5	15	A	Geranyl acetate/Acetophenone
		B	Pentyl acetate/ 2,6-Dimethoxy-phenol
*Vir*ab6 (like *Mel*ab6)	21	A	1-octen-3-ol
		B	Guaiacol
*Vir*ab7	26	A	6-Methyl-5-hepten-2-one/1-octen-3-ol
		B	2-Heptanone
*Vir*ab8	13	A	1-Hexanol
		B	Isopropyl propionate/Ethyl lactate
*Vir*ab9	15	A	Linalool
		B	Methyl benzoate
*Vir*ab10	24	A	Geranyl acetate/Acetoin
		B	Ethyl-3-hydroxybutyrate
*Vir*ab11	16	A	Ethyl benzoate
		B	Isopropyl benzoate

Supplementary table 7: Two-way analysis of variance table showing the effect of the developmental (F1) and experimental (F2) temperature on the valence value of guaiacol, methyl salicylate, and isopropyl benzoate in *D. ezoana*, *D. novamexicana*, and *D. virilis*.

		Factors	Df	Sum Sq	Mean Sq	F value	Pr(>F)
D. ezoana	guaiacol	F1	1	0.141	0.1412	0.659	**0.042**
		F2	3	7.34	2.4468	11.421	***P<0.0001***
		F1 ×F2	3	0.356	0.1187	0.554	0.647
	methyl salicylate	F1	1	0.042	0.0424	0.196	**0.0065**
		F2	3	3.29	1.0968	5.058	**0.0031**
		F1 ×F2	3	0.263	0.0876	0.404	0.7505
	isopropyl benzoate	F1	1	0.224	0.22372	1.232	0.271
		F2	3	0.079	0.02634	0.145	0.933
		F1 ×F2	3	0.743	0.24781	1.364	0.261
D. novamexicana	guaiacol	F1	1	0.283	0.2827	0.96	**0.033**
		F2	3	0.862	0.2873	0.976	**0.0409**
		F1 ×F2	3	0.846	0.282	0.958	0.417
	methyl salicylate	F1	1	0.01	0.0104	0.063	**0.0028**
		F2	3	0.562	0.1874	1.13	**0.03426**
		F1 ×F2	3	1.965	0.6549	3.95	**0.0115**
	isopropyl benzoate	F1	1	0.049	0.049	0.243	0.623
		F2	3	1.03	0.3435	1.705	0.174
		F1 ×F2	3	0.223	0.0743	0.369	0.776
D. virilis	guaiacol	F1	1	0.147	0.1474	1.296	0.25875
		F2	3	1.77	0.59	5.187	**0.00267**
		F1 ×F2	3	0.491	0.1637	1.439	0.23844
	methyl salicylate	F1	1	0.051	0.05105	0.33	0.568
		F2	3	0.614	0.20456	1.321	**0.0274**
		F1 ×F2	3	0.503	0.16782	1.084	0.362
	isopropyl benzoate	F1	1	0.008	0.008	0.043	0.837
		F2	3	0.384	0.1279	0.675	0.57
		F1 ×F2	3	0.976	0.3253	1.719	0.171

Number in bold/italic depict significant differences

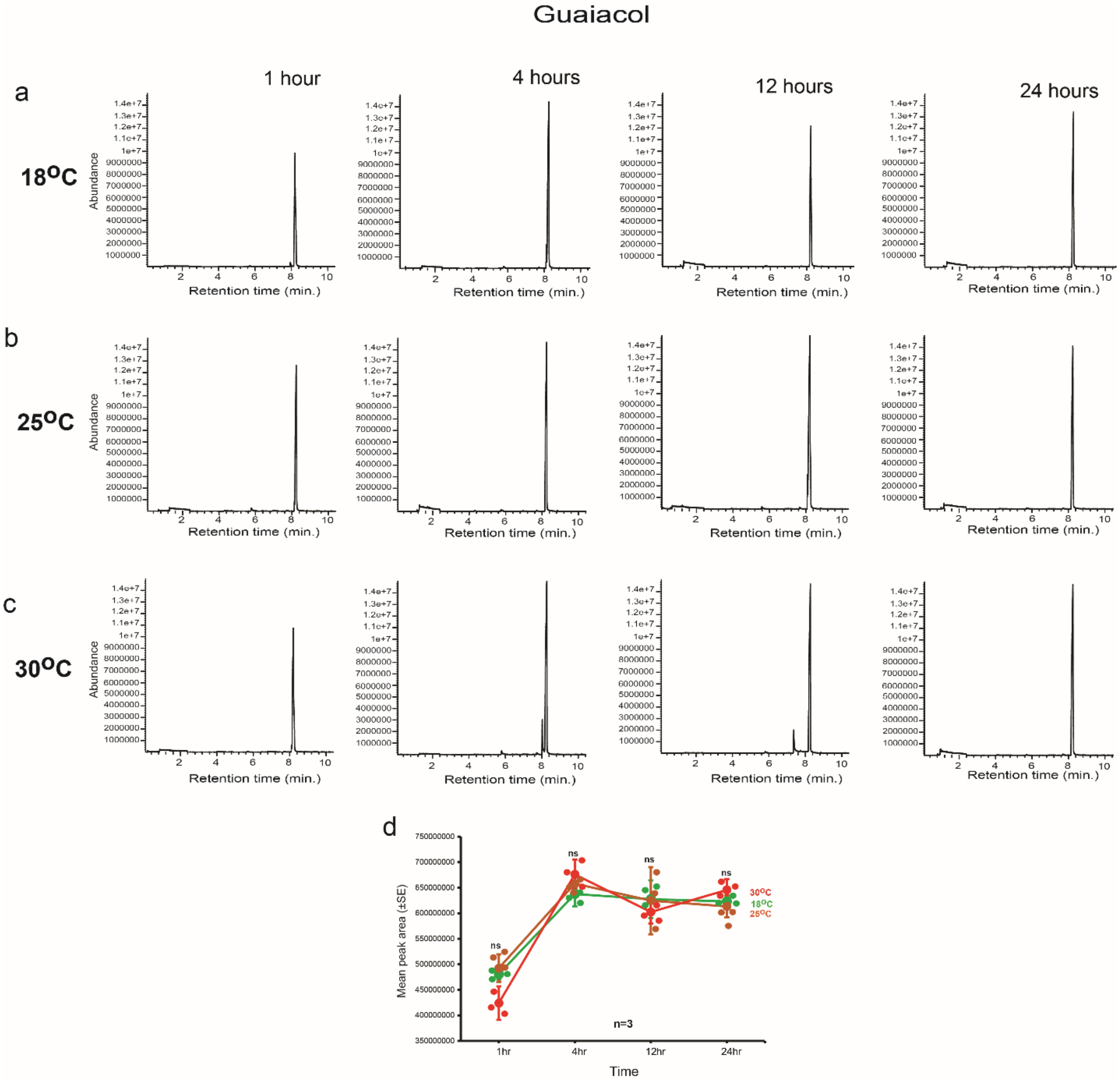

Supplementary figure 7. **Release rate of guaiacol in function of temperature and time.** Representative GC-MS chromatogram of guaiacol (10^{-2}) when exposed to **(a)** 18°C, **(b)** 25°C and **(c)** 30°C for 1, 4, 12 and 24 hours. **(d)** Line graphs illustrating the change of peak area of guaiacol over time when kept at 18, 25 and 30°C. Error bars indicate standard error of the mean (SEM)

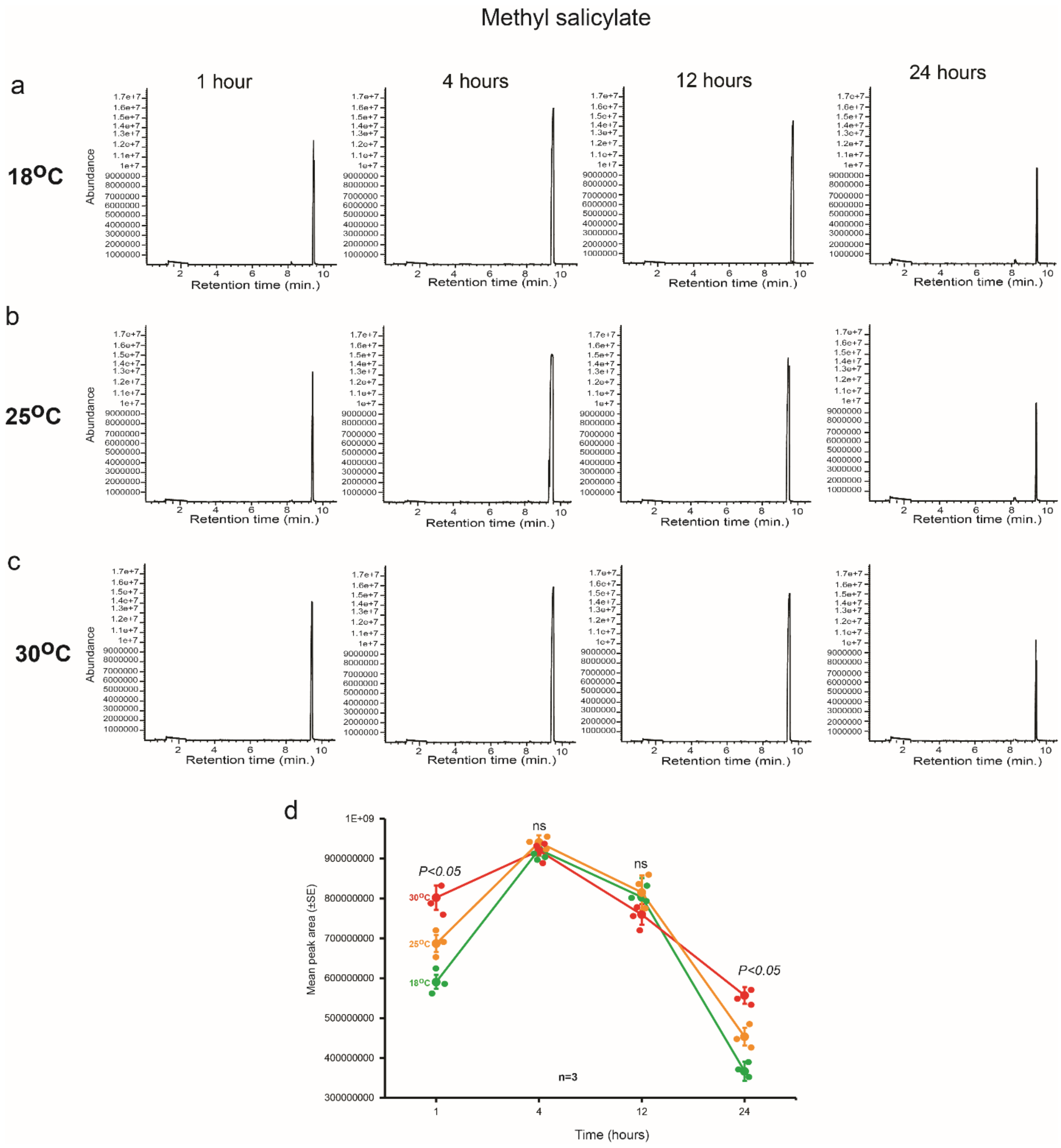

Supplementary figure 8. Release rate of Methyl salicylate in function of temperature and time. Representative GC-MS chromatogram of Methyl salicylate (10^{-2}) when exposed to **(a)** 18°C, **(b)** 25°C and **(c)** 30°C for 1, 4, 12 and 24 hours. **(d)** Line graphs illustrating the change of peak area of methyl salicylate over time when kept at 18, 25 and 30°C. Error bars indicate standard error of the mean (SEM). Error bars indicate standard error of the mean (SEM)
