# Supplementary material for: Temperature-dependent modulation of odor-dependent behavior in three drosophilid fly species of differing thermal preference
Source: Commun Biol. 2023 Sep 4;6:905. doi: 10.1038/s42003-023-05280-5 (PMC10477191; doi:10.1038/s42003-023-05280-5)
Supplement: Supplementary file 4 — Reporting Summary [file 42003_2023_5280_MOESM4_ESM.pdf]

nature portfolio

Corresponding author(s): Bill S Hansson

Last updated by author(s): Apr 20, 2023

Reporting Summary

Nature Portfolio wishes to improve the reproducibility of the work that we publish. This form provides structure for consistency and transparency in reporting. For further information on Nature Portfolio policies, see our Editorial Policies and the Editorial Policy Checklist.

Statistics

For all statistical analyses, confirm that the following items are present in the figure legend, table legend, main text, or Methods section.

n/a	Confirmed	
☐	☒	The exact sample size (n) for each experimental group/condition, given as a discrete number and unit of measurement
☐	☒	A statement on whether measurements were taken from distinct samples or whether the same sample was measured repeatedly
☐	☒	The statistical test(s) used AND whether they are one- or two-sided<br>*Only common tests should be described solely by name; describe more complex techniques in the Methods section.*
☒	☐	A description of all covariates tested
☐	☒	A description of any assumptions or corrections, such as tests of normality and adjustment for multiple comparisons
☐	☒	A full description of the statistical parameters including central tendency (e.g. means) or other basic estimates (e.g. regression coefficient) AND variation (e.g. standard deviation) or associated estimates of uncertainty (e.g. confidence intervals)
☐	☒	For null hypothesis testing, the test statistic (e.g. F, t, r) with confidence intervals, effect sizes, degrees of freedom and P value noted<br>*Give P values as exact values whenever suitable.*
☒	☐	For Bayesian analysis, information on the choice of priors and Markov chain Monte Carlo settings
☒	☐	For hierarchical and complex designs, identification of the appropriate level for tests and full reporting of outcomes
☒	☐	Estimates of effect sizes (e.g. Cohen's d, Pearson's r), indicating how they were calculated

Our web collection on statistics for biologists contains articles on many of the points above.

Software and code

Policy information about availability of computer code

Data collection	Thermal preference and trap assay data were collected using visual observations. Electrophysiology data were collected using the Auto Spike software (Syntech, version 3.7)
Data analysis	All statistical analyses were performed using freely accessible software, R version 4.0.3 (R Core Team, 2020) and PAST version 3.09

For manuscripts utilizing custom algorithms or software that are central to the research but not yet described in published literature, software must be made available to editors and reviewers. We strongly encourage code deposition in a community repository (e.g. GitHub). See the Nature Portfolio guidelines for submitting code & software for further information.

Data

Policy information about availability of data

All manuscripts must include a data availability statement. This statement should provide the following information, where applicable:

- Accession codes, unique identifiers, or web links for publicly available datasets
- A description of any restrictions on data availability
- For clinical datasets or third party data, please ensure that the statement adheres to our policy

Provide your data availability statement here.

Research involving human participants, their data, or biological material

Policy information about studies with human participants or human data. See also policy information about sex, gender (identity/presentation), and sexual orientation and race, ethnicity and racism.

Reporting on sex and gender	No applicable
Reporting on race, ethnicity, or other socially relevant groupings	No applicable
Population characteristics	No applicable
Recruitment	No applicable
Ethics oversight	No applicable

Note that full information on the approval of the study protocol must also be provided in the manuscript.

Field-specific reporting

Please select the one below that is the best fit for your research. If you are not sure, read the appropriate sections before making your selection.

☒ Life sciences ☐ Behavioural & social sciences ☐ Ecological, evolutionary & environmental sciences

For a reference copy of the document with all sections, see nature.com/documents/nr-reporting-summary-flat.pdf

Life sciences study design

All studies must disclose on these points even when the disclosure is negative.

Sample size	A sample size of 30 is common across statistics. We largely exceeded this in all the experiments. For the thermal preference we used 50 flies for each treatment. In the SSR assays, we sampled 200 individual basiconics in each species . We tested 10 groups of 20 flies (200 flies) in the trap assays
Data exclusions	No data were excluded from the analysis
Replication	As well as indicating on each figure caption the number of replicates used, we showed on each figure, the individual replicate (dot point)
Randomization	All flies used in the study were collected from different vials and generations to ensure randomization.
Blinding	Flies of the same age from different vials were always mixed before being used for experimentation

Reporting for specific materials, systems and methods

We require information from authors about some types of materials, experimental systems and methods used in many studies. Here, indicate whether each material, system or method listed is relevant to your study. If you are not sure if a list item applies to your research, read the appropriate section before selecting a response.

Materials & experimental systems

n/a	Involved in the study	
☒	☐	Antibodies
☒	☐	Eukaryotic cell lines
☒	☐	Palaeontology and archaeology
☐	☒	Animals and other organisms
☒	☐	Clinical data
☒	☐	Dual use research of concern
☒	☐	Plants

Methods

n/a	Involved in the study	
☒	☐	ChIP-seq
☒	☐	Flow cytometry
☒	☐	MRI-based neuroimaging

Animals and other research organisms

Policy information about studies involving animals; ARRIVE guidelines recommended for reporting animal research, and Sex and Gender in Research

Laboratory animals	Drosophila ezoana (stock number: E-15701) , D. novamexicana (stock number: 15010-1031.08), D. virilis (stock number: 15010-1051.00)
Wild animals	No wild animals were tested
Reporting on sex	We considered sex as a factor when analyzing data from the temperature preference assay. The proportions of males and females that made (or did not) the choice were similar, we therefore excluded this factor when analyzing data from the traps assays.
Field-collected samples	No samples were collected from the field
Ethics oversight	All experiments were conducted on flies and did not necessitate ethical approval

Note that full information on the approval of the study protocol must also be provided in the manuscript.
